# Supplementary material for: Intracellular functions and motile properties of bi-directional kinesin-5 Cin8 are regulated by neck linker docking
Source: eLife. 2021 Aug 13;10:e71036. doi: 10.7554/eLife.71036 (PMC8456603; doi:10.7554/eLife.71036)
Supplement: Supplementary file 1. [file elife-71036-supp1.docx]

**List of plasmids used in this study**

| **Plasmid** | **Genotype** |
| --- | --- |
| pMA1208  (Gheber et al., 1999) | *CIN8, CYH2, LEU2, CEN* |
| pVF68 | *CIN8-3EGFP, URA3, CEN* |
| pLG73 | *Cin8_NL_KHC-3EGFP, URA3, CEN* |
| pLG74 | *Cin8_NL_Eg5-3EGFP, URA3, CEN* |
| pLG75 | *Cin8-G522N-3EGFP, URA3, CEN* |
| pLG76 | *Cin8-M526T-3EGFP, URA3, CEN* |
| pLG77 | *Cin8-Q520E-3EGFP, URA3, CEN* |
| pLG80 | *Cin8_NL_Eg5-N522G-3EGFP, URA3, CEN* |
| pLG81 | *Cin8-D528K-3EGFP, URA3, CEN* |
| pLG82 | *Cin8-K516M-3EGFP, URA3, CEN* |
| pLG83 | *Cin8_NL_Cut7-3EGFP, URA3, CEN* |
| pOS7 | *P_GAL1_-CIN8-TEV-EGFP-6His, LEU2, 2*µ |
| pSAG37 | *P_GAL1_Cin8_NL_Eg5-TEV-EGFP-6His, LEU2, 2*µ |
| pSAG38 | *P_GAL1_Cin8-G522N-TEV-EGFP-6His, LEU2, 2*µ |
| pSAG49 | *P_GAL1_Cin8_NL_Eg5-N522G-TEV-EGFP-6His, LEU2, 2*µ |
| pSAG50 | *P_GAL1_Cin8_NL_Cut7-TEV-EGFP-6His, LEU2, 2*µ |
| pRS316 | *URA3, CEN* |
| pKA1 | *CIN8-3HA, URA3, CEN* |
| pKA2 | *Cin8_NL_Eg5-N522G-3HA, URA3, CEN* |
| pKA3 | *Cin8-G522N-3HA, URA3, CEN* |
| pKA4 | *Cin8_NL_Cut7-3HA, URA3, CEN* |
| pKA5 | *Cin8_NL_Eg5-3HA, URA3, CEN* |
