# Supplementary material for: Intracellular functions and motile properties of bi-directional kinesin-5 Cin8 are regulated by neck linker docking
Source: eLife. 2021 Aug 13;10:e71036. doi: 10.7554/eLife.71036 (PMC8456603; doi:10.7554/eLife.71036)
Supplement: Supplementary file 2. [file elife-71036-supp2.docx]

***Saccharomyces cerevisiae* strains used in this study**

| **Yeast strain** | **Genotype** | **Experiment** |
| --- | --- | --- |
| LGY 620 | *MATa, ura3-52, leu2-3,112, his3-Δ200, lys2-801, ade2-101, cyh2^r^, cin8::HIS3, kip1::HIS3, (pMA1208: CIN8, CYH2, LEU2, CEN)* | Cell viability and doubling time |
| LGY 727 | *MATa, ura3-52, leu2-3,112, his3-Δ200, lys2-801, ade2-101. cin8::LEU2* | Doubling time in the presence of Kip1 |
| LGY 1694 | *MATα, ura3-52, leu2-3,112, pep4-3, prb1-1122, reg1-501, gal1* | Overexpression of Cin8 NL variants for motility assays |
| LGY 3989 | *MATa, ura3-52, leu2-3,112, his3-Δ200, lys2-801, ade2-101. cin8::LEU2, SPC42::Spc42-tdTomato, kanMX* | Live cell imaging |
